# Genome-Wide Analysis of the *IQM* Gene Family in Rice (*Oryza sativa* L.)

**DOI:** 10.3390/plants10091949

**Published:** 2021-09-18

**Authors:** Tian Fan, Tianxiao Lv, Chuping Xie, Yuping Zhou, Changen Tian

**Affiliations:** Guangzhou Key Laboratory for Functional Study on Plant Stress-Resistant Genes, School of Life Sciences, Guangzhou University, Guangzhou 510006, China; ftt860630@163.com (T.F.); ljl_vv@126.com (T.L.); xiechuping@gzhu.edu.cn (C.X.); zhouxp@gzhu.edu.cn (Y.Z.)

**Keywords:** rice, *IQM* genes, expression pattern, IQ motif

## Abstract

Members of the *IQM* (IQ-Motif Containing) gene family are involved in plant growth and developmental processes, biotic and abiotic stress response. To systematically analyze the *IQM* gene family and their expression profiles under diverse biotic and abiotic stresses, we identified 8 *IQM* genes in the rice genome. In the current study, the whole genome identification and characterization of *OsIQMs*, including the gene and protein structure, genome localization, phylogenetic relationship, gene expression and yeast two-hybrid were performed. Eight *IQM* genes were classified into three subfamilies (I–III) according to the phylogenetic analysis. Gene structure and protein motif analyses showed that these *IQM* genes are relatively conserved within each subfamily of rice. The 8 *OsIQM* genes are distributed on seven out of the twelve chromosomes, with three *IQM* gene pairs involved in segmental duplication events. The evolutionary patterns analysis revealed that the *IQM* genes underwent a large-scale event within the last 20 to 9 million years. In addition, quantitative real-time PCR analysis of eight *OsIQMs* genes displayed different expression patterns at different developmental stages and in different tissues as well as showed that most *IQM* genes were responsive to PEG, NaCl, jasmonic acid (JA), abscisic acid (ABA) treatment, suggesting their crucial roles in biotic, and abiotic stress response. Additionally, a yeast two-hybrid assay showed that OsIQMs can interact with OsCaMs, and the IQ motif of OsIQMs is required for OsIQMs to combine with OsCaMs. Our results will be valuable to further characterize the important biological functions of rice *IQM* genes.

## 1. Introduction

As one of the most important intracellular second messenger, Ca^2+^ plays a prominent role in many essential biological processes of plants, such as numerous developmental processes and responses to various kinds of biotic and abiotic stresses [1]. The spatial and transient changes of cytoplasmic Ca^2+^ levels in response to various stresses were detected and decoded by specific Ca^2+^ sensors, which then transduce these changes of Ca^2+^ levels into a series of downstream effects [2,3].

To date, most of the Ca^2+^ sensors have been identified to contain conserved Ca^2+^-binding helix-loop-helix EF-hand motif [4]. Based on the number of EF hand motifs, four types of Ca^2+^ sensors were identified in higher plants: calmodulins (CaM), CaM-like proteins (CML), calcineurin B-like proteins (CBL) and calcium-dependent protein kinases (CDPK) [5,6]. Among these Ca^2+^ sensors, CaM is a highly conserved small, acidic protein and has been getting the most attention in Ca^2+^ signal transduction [7]. CaM has no enzymatic activity of its own; however, CaM produces a change in conformation after binding Ca^2+^, and then it can activate a wide range of target proteins named calmodulin-binding proteins (CaMBPs) involved in diverse cellular processes [7,8].

The CaMBPs include transcription factors, metabolic enzymes, cytoskeleton, chaperones and kinases to involve in plant development, metabolic regulation, stress response, defense reactions [1,9]. The recognition motifs for the interaction between CaM and CaMBPs contain three conserved types: one is an IQ motif [ILV] QxxxRxxxx [R, K] or (IQxxxRGxxxR), which binds CaM in a Ca^2+^-independent manner, others are the 1-5-10 motif ([FILVW] × 3[FILV] × [FILVW]) and 1-8-14 motif ([FILVW] × 6[FAILVW] × 5[FILVW]), which are thought to mediate CaM retention in a Ca^2+^-dependent manner [10]. In plants, five classes of the IQ motif-containing protein family, namely, the IQ67-domain containing protein (IQD) family, the myosin protein family, the calmodulin-binding transcription activator (CAMTA) family, the cyclic nucleotide-gated channels (CNGC) family and the IQ motif containing protein (IQM) family, have been identified [11]. The members of these five families had divergence in the number and distribution of IQ motifs. Compared to other families, the functional studies of the IQM family are needed to explore more. To date, the plant-specific *IQM* gene family have been comprehensively analyzed only in Arabidopsis genomes. In Arabidopsis, six members named IQM1 to IQM6 were identified [11]. Across all IQM gene family members, only one IQ motif, located in the N-terminal of these proteins, is the common characteristic. Moreover, the IQ motif overlaps with the sequence, which is similar to the pea heavy-metal induced protein 6A (PHMIP 6A) [12]. Meanwhile, there is a segment of trichosanthin that consists of 248 amino-acid residues in the C-terminal [11]. Subsequently, a few functional investigations of IQM proteins have been reported in recent years: in Arabidopsis thaliana, AtIQM1 proteins can interact with AtCaM5 in a Ca^2+^-dependent manner and modulates stomatal movement by affecting the content of ROS [13]. Furthermore, it was reported that AtIQM1 is a key regulatory factor in plant defense against the necrotrophic pathogen Botrytis cinerea by regulating JA biosynthesis [14]. In addition, AtIQM4 is involved in seed dormancy and germination [15], and the mutation of AtIQM5 delays flowering possibly through modulating the juvenile-to-adult transition [16]. Although, some previous studies had revealed the roles of some IQMs and the underlying mechanisms in Arabidopsis. On the other hand, the understanding and systematic analysis of the IQM family members in the other crops need to be explored. We considered that the *IQM* gene family might have similar functions in rice as in Arabidopsis. Therefore, to obtain a better understanding of the *IQM* gene family and to improve future study of this family with regard to resistance to biotic and abiotic stress in rice, the current study performed a comprehensive genome-wide analysis of them. In this study, eight non-redundant *IQM* encoding genes were identified by genome-wide analysis technology. The phylogenetic relationships, gene structure, conserved motifs, evolutionary patterns and divergence, yeast two-hybrid assay and expression profiling in response to abiotic stress and hormone were explored, providing a theoretical foundation for the downstream functional analysis of the rice *IQM* gene.

## 2. Results

### 2.1. Identification of IQM Gene Family in Rice Genome

To identify the *IQM* gene family in rice, the previously reported Arabidopsis IQM proteins and their IQ domain were used as the query sequences to search the RAP-DB (http://rapdb.dna.affrc.go.jp/ accessed on 19 July 2020). Here, a total of eight putative *OsIQM* genes contained the conserved IQ calmodulin-binding motif were identified through SMART. The present study coined these eight *OsIQM* genes as *OsIQM1* to *OsIQM8*, according to their physical locations (from top to bottom) on chromosomes 1–12 (Table 1). The characteristics of the *OsIQM* genes, including ORFs, amino acids (aa), isoelectric point (pI), molecular weight (MW), chromosome location and start position, were listed in Table 1. The identified *OsIQM* gene family members encode proteins ranging from 475 to 650 aa in length and the predicted molecular masses ranged from 52.2 to 73.1 kDa. Additionally, the predicted pI values ranged from 6.19 to 9.28. 

### 2.2. Phylogenetic Analysis of the OsIQM Gene Family

**Figure 1:**
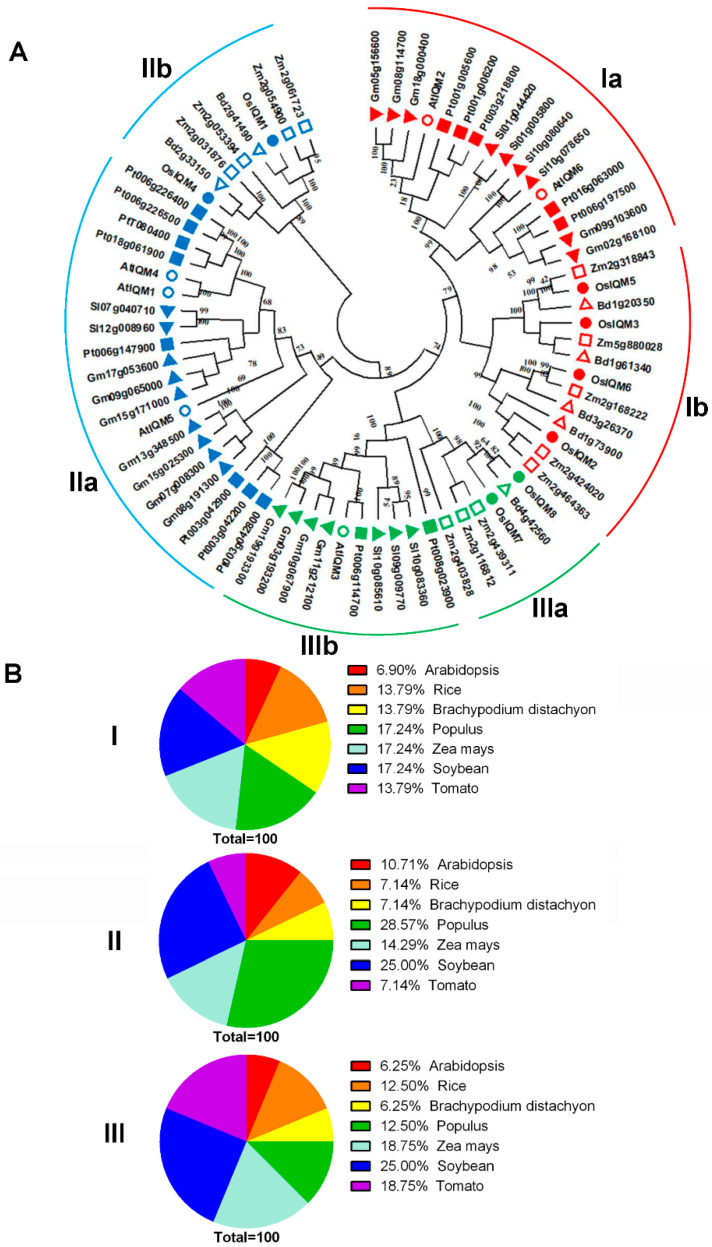
Phylogenetic tree and distribution of IQM protein from 7 plant species. (**A**) The full-length amino acid sequences of the IQM proteins from *Brachypodium distachyon*, *Oryza sativa*, *Arabidopsis thaliana*, *Populus trichocarpa*, *Zea mays*, *Glycine max* and *Solanum lycopersicum* were aligned using ClustalX 1.83, and the phylogenetic tree was constructed by the NJ method with MEGA X. The number of bootstrap values was 1000 replicates. Red—subfamily I; blue—subfamily II; green—subfamily III. IQM proteins from the same species were marked with geometrical patterning: hollow circle—*Arabidopsis thaliana*; filled circle—*Oryza sativa*; hollow triangle—*Brachypodium distachyon*; filled inverse-triangle—*Glycine max*; filled triangle—*Solanum lycopersicum*; filled quadrate—*Populus trichocarpa*; hollow quadrate—*Zea mays*. (**B**) A percentage representation of IQM proteins across the plant species in each subfamily.

To better understand the molecular evolution and phylogenetic relationship, the current study employed MEGA X software to establish the unrooted phylogenetic tree of IQM members from *Brachypodium distachyon*, *Oryza sativa*, *Arabidopsis thaliana*, *Populus trichocarpa*, *Zea mays*, *Glycine max* and *Solanum lycopersicum* [17]. A phylogenetic tree from the alignment of full-length IQM protein sequences was constructed using the neighbor-joining method. The NJ phylogenetic tree contained 73 IQM protein sequences from *Oryza sativa* (8), *Brachypodium distachyon* (7), *Arabidopsis thaliana* (6), *Populus trichocarpa* (15), *Zea mays* (12), *Glycine max* (16) and *Solanum lycopersicum* (9). The results showed that these IQMs could be separated into three different subfamilies (I, II, and III) (Figure 1A). The IQM I and II subfamilies covered mostly *IQM* genes. By contrast, the IQM III subfamily had the fewest *IQM* genes (Figure 1A). Generally, subfamily I and II constituted the most clade, containing 29 and 28 IQMs, which accounted for 50% and 48% of the total IQM genes, respectively. We further examined each of the subfamilies and found they all separated into two distinct groups (a and b) of monocot and eudicot (Figure 1A). This result suggested the IQM proteins in monocot and eudicot may produce different functions. Additionally, as shown in Figure 1B, PtIQMs, ZmIQMs and GmIQMs account for 17.24% in subfamily I; PtIQMs account for 28.57% in subfamily II; GmIQMs account for 25% in subfamily III. It revealed that the plant IQM protein sequence distribution predominates with species bias and there were more *IQM* genes in each subfamily for dicotyledonous than for monocotyledonous.

In the phylogenetic tree, the putative orthologous genes of *OsIQMs* were found in *Zea mays* and *Brachypodium distachyon*, which supported by the three ortholog pairs (*OsIQM1/Bd2g41490*, *OsIQM4/Bd2g33150*, *OsIQM5/Zm2g318843*, *OsIQM6/Zm2g168222* and *OsIQM8/Bd4g42560*) (Figure 1A). There were three monocot ortholog pairs that were found in *Oryza sativa*/*Brachypodium distachyon.* Only one eudicot ortholog pairs were observed in *Arabidopsis* *thaliana* and *Populus trichocarpa* (Figure 1A). Generally, the monocot *IQM* genes share branches with the nearest monocot orthologs, while eudicot *IQM* genes form sister pairs with the nearest eudicot orthologs. Indeed, the ortholog pair developed by eudicot and monocot was not found in the phylogenetic tree (Figure 1A), indicating the member of each subfamily from eudicot and monocot may not come from a common ancestor.

### 2.3. Gene Structure and Conserved Motifs of OsIQM Genes

**Figure 2:**
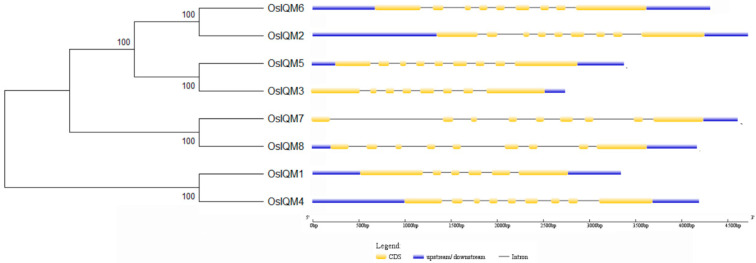
Phylogenetic relationship and exon–intron organization of rice *IQM* genes. The unrooted phylogenetic tree of eight rice IQM proteins was constructed by the NJ method with 1000 bootstrap replicates. Exons and introns are represented by yellow boxes and black lines, respectively. Untranslated regions (UTRs) are indicated by blue boxes. The sizes of each *IQM* gene can be estimated using the scale at the bottom.

In order to understand the structural diversity of rice *IQM* genes, we first constructed an unrooted phylogenetic tree based on the alignment of the full-length sequences of the eight OsIQM proteins, and sequences of rice IQM proteins were retrieved from the RAP-DB database (http://rapdb.dna.affrc.go.jp/ accessed on 19 July 2020). The rice IQM family members can be classified into three subfamilies: subfamily I, II and III, with each subfamily containing four, two, and two members, respectively (Figure 2). These results were in good agreement with that described above for the 7 plant species (Figure 1A and Figure 2). These eight *OsIQM* genes formed four sister pairs, and all of them displayed high bootstrap support. The exon–intron organization map analysis indicated that the eight *OsIQM* genes contain different numbers of exons, ranging from six to nine (Figure 2). Furthermore, the genes in one subfamily shared similar gene structures in terms of either the intron/exon number or length. However, one sister gene pairs showed greater changes in their structural organizations (*OsIQM1/4*) and varying numbers of exons and introns.

**Figure 3:**
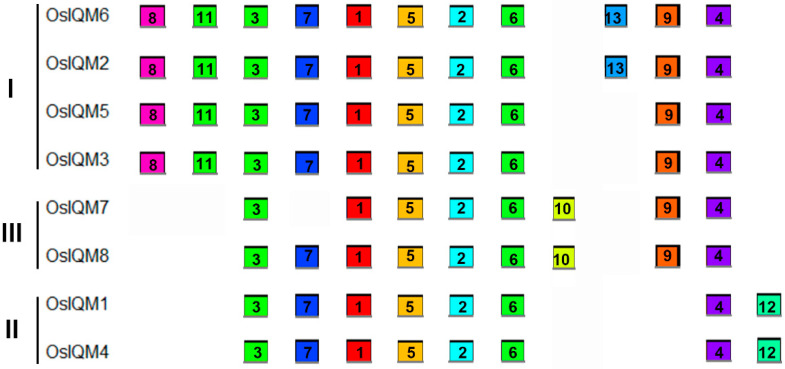
Motif distribution in IQM proteins of rice. Motifs of the OsIQM proteins were identified by the online MEME program. Each motif was represented by a different colored box with its serial number in central of the box. The colored boxes were ordered manually according to the results of the MEME server. The length of each colored box does not represent the actual motif size in the corresponding proteins.

MEME was used to analyze protein motifs in the eight IQM protein sequences in rice (Figure 3). In total, 13 conserved motifs were identified and named motif 1–13. Figure S2 presents the conserved amino acid sequences and the length of each motif. Each of the putative motifs identified by MEME was subsequently verified using the Pfam and SMART. Among them, motif three was found to encode the IQ domain and related to heavy metal-induced protein 6 (HMIP6) of pea [11], motif five was considered to have similarity with trichosanthin [11], while motif six is a representative JmjN motif. However, the biological annotation of the other putative motifs remains unclear and need to be further investigated. IQM proteins in the same subfamily often have a common motif, suggesting functional similarities among these IQM proteins. In addition, some motifs were also detected in the specific subfamily. For example, motifs 12 were only observed in the subfamily II and motifs 10 are only present in subfamily III (Figure 3). These subfamily-specific motifs may produce functional divergence of *IQM* genes in rice.

### 2.4. Chromosomal Locations and Gene Duplication

**Figure 4:**
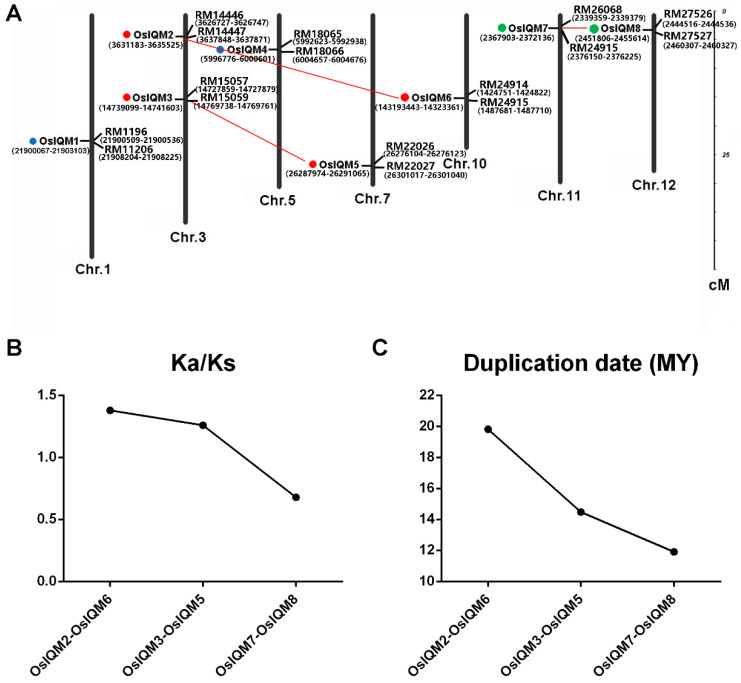
Chromosomal distribution and segmental duplication events of *IQM* genes in rice. (**A**) Eight *OsIQM* genes are mapped to 7 of the 12 rice chromosomes. The nearest SSR markers of eight *OsIQM* genes were mapped to 7 rice chromosomes. The duplicated paralogous pairs of *OsIQM* genes were connected with red lines. Chromosome numbers were located at the bottom of each vertical bar. Different colored circles on the left of the gene name represented corresponding subfamily which this gene belongs to. Red—subfamily I; blue—subfamily II; green—subfamily III. (**B**,**C**) Distribution of Ka/Ks values (**B**) and duplication date (MY) (**C**) were obtained from paralogous gene-pairs in the rice genome.

The distribution of *OsIQM* genes was constructed by their positions in the rice chromosomes. The results show that eight *OsIQM* genes are unevenly distributed across 7 of the 12 chromosomes (Figure 4A). Both *OsIQM2* and *OsIQM3* were localized to chromosome 3. *OsIQM1*, *OsIQM4*, *OsIQM5*, *OsIQM6*, *OsIQM7* and *OsIQM8* were localized on chromosomes 1, 5, 7, 10, 11 and 12, respectively (Figure 4A). The nearest SSR markers of eight *OsIQM* genes were mapped to 7 rice chromosomes. Segmental duplication, tandem duplication and retroposition have been suggested to drive the expansion of gene families in genomes [18]. To evaluate the evolutionary patterns of the *OsIQM* gene family, segmental duplication and tandem duplication analysis were performed. According to the rice segmental duplication database from RGAP, we found three pairs (*OsIQM2/OsIQM6*, *OsIQM3/OsIQM5* and *OsIQM7/OsIQM8*) that were located in duplicated chromosomal regions, suggesting that six genes came from segmental duplication. In addition, the chromosome distribution analysis indicated that tandem duplications have not been involved in the expansion of the *OsIQM* gene. These results indicated that the expansion of the *OsIQM* gene family results from segmental duplication events.

To explore the evolutionary selection of the three pairs of *OsIQM* genes, the Ka (non-synonymous substitution rate), the Ks (synonymous substitution rate) and the Ka/Ks ratios were calculated for each duplicated *OsIQM* gene pair in the family. As we know, Ka/Ks ratio < 1 indicates purifying selection which eliminates deleterious mutations and maintains the protein, where the Ka/Ks ratio > 1 indicates positive selection that accelerated evolution by changing the protein [19]. Our study showed that the Ka/Ks ratio for the one segmentally duplicated gene pair (*OsIQM7/OsIQM8*) was <1, suggesting that the gene pair had mainly had negative or purifying selection after the duplication events. The Ka/Ks ratios of *OsIQM2/OsIQM6* and *OsIQM3/OsIQM5* were more than 1, suggesting that the two duplicated pairs had evolved under positive selection (Figure 4B). According to the neutral evolutionary rate (λ) of 6.5 × 10^−9^ substitutions per synonymous site per year for the rice genome, we calculated the dates of the duplication events [20]. The divergence time of duplicated *OsIQM* gene pairs ranged from 11.9 to 19.8 Mya (Figure 4C). The duplication time within the last 20 to 9 million years for the three pairs occurred after *Zizaniinae* and *Oryzinae* were divided and before the *Oryza* genus branched off from the remaining genera of *Oryzeae* [21].

### 2.5. The Subcellular Localization of OsIQMs

**Figure 5:**
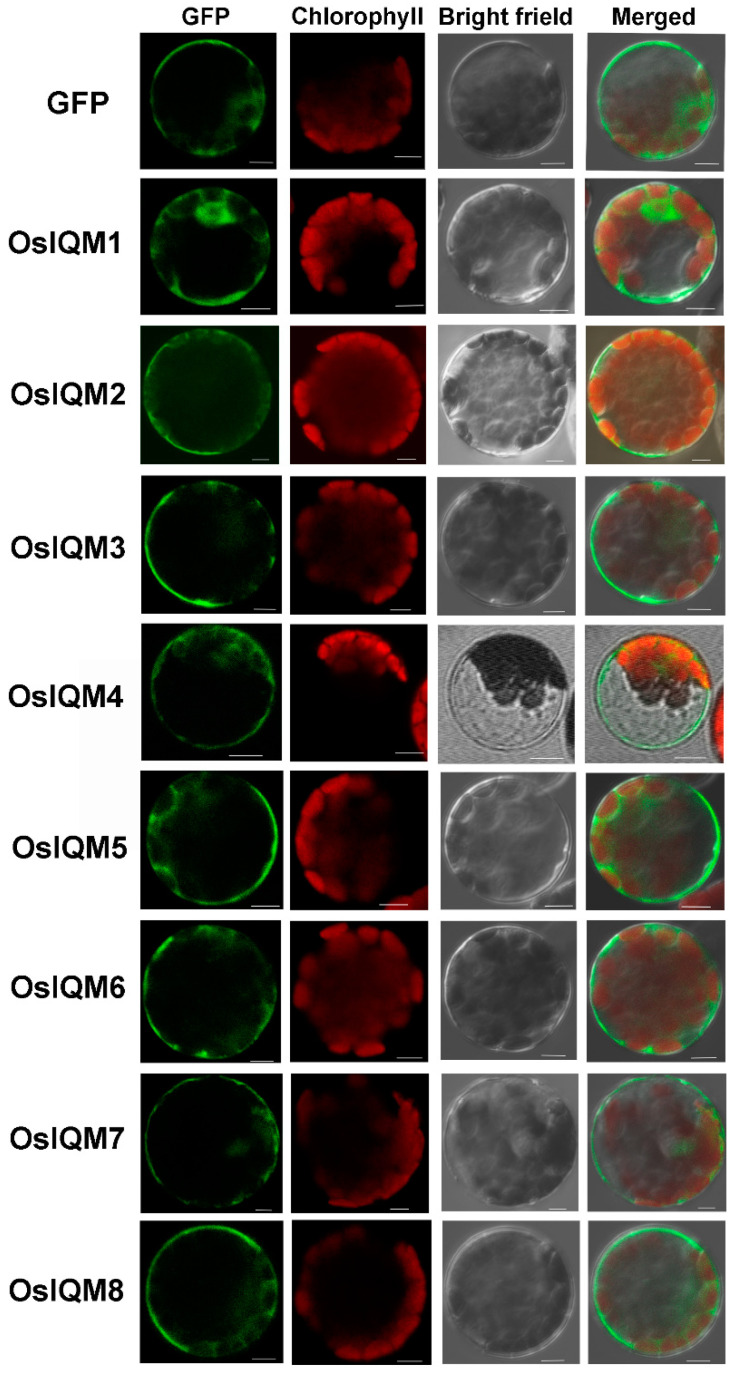
Subcellular localization of OsIQMs in Arabidopsis protoplasts. Bars = 5 μm. Bright, bright-field image; GFP—GFP fluorescence image; Chlorophyll—chlorophyll autofluorescence; merged—merged bright-field, GFP fluorescence and chlorophyll autofluorescence images.

In order to gain further insights into these OsIQM proteins, we predict their possible subcellular localization patterns by WoLF PSORT. The WoLF PSORT prediction analysis indicated that OsIQM2–8 are localized in the cell nucleus and OsIQM1 are localized in the chloroplasts (Table 1). To further determine the subcellular localization of OsIQMs, full-length cDNA was fused with Green Fluorescent Protein (GFP) driven by the CMV 35S promoter and transiently expressed in protoplasts of Arabidopsis cells. The results showed the OsIQM1 was localized in both the cytosol and nucleus, while the OsIQM2–8 were mainly localized in the cytosol. (Figure 5). These indicated the localization of OsIQMs is not consistent with the predicted localization using WoLF PSORT programs.

### 2.6. Protein Interaction Analysis of OsIQMs with OsCaMs

**Figure 6:**
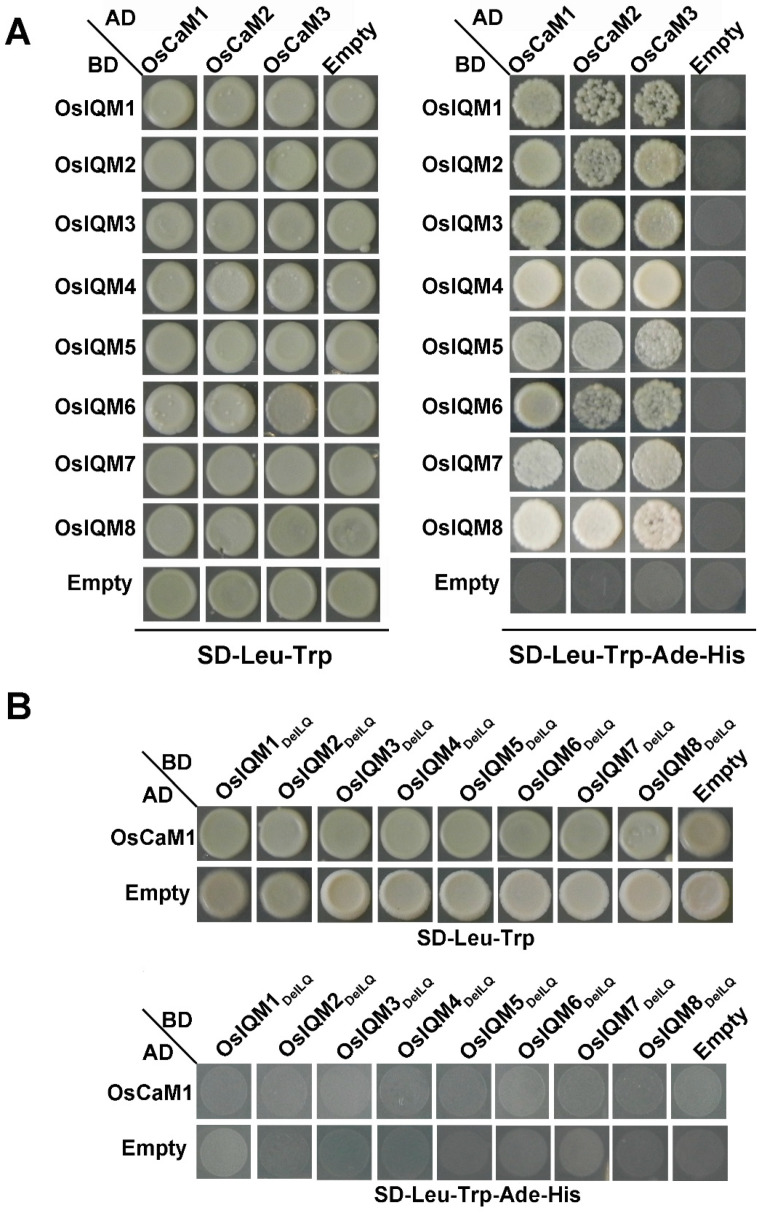
OsIQMs interaction with OsCaMs and exploration of the calmodulin-binding site of OsIQMs. (**A**) The bait construct of pGBKT7-OsIQMs and prey construct were co-transformed into the yeast strain AH109 and then were examined on SD/-Trp/-Leu and SD/-Trp/-Leu/-His/-Ade plates. (**B**) The interaction between OsCaM1 and OsIQMs_DelLQ_. Positive control: pGBKT7-53 and pGADT7-T; Negative control: pGBKT7-Lam and pGADT7-T.

Previous studies have shown that IQM1 can interact with CaM5 in *Arabidopsis* [13]. To verify whether this interaction also exists in rice, we used yeast two-hybrid analysis to detect the interaction between OsIQMs with OsCaMs. Our results showed that AD-OsCaMs (OsCaM1, 2, 3) and BD-OsIQMs (OsIQM1, 2, 3, 4, 5, 6, 7, 8) co-transform into AH109 yeast cells, and all the transformants were found to grow well on SD/-Trp/-Leu/-Ade/-His medium, indicating that OsIQMs and OsCaMs interacted in yeast (Figure 6A).

### 2.7. Mutations in the IQ Motif of OsIQMs Result in the Loss of Affinity CaM Binding

In Arabidopsis, the IQ motif in AtIQM1 was identified to bind with CaM [13]. In order to verify whether the IQ motif can bind with CaM in rice, we used yeast two-hybrid analysis to examine the interaction between OsIQMs_DelLQ_ with OsCaM1. Our results showed that when the recombinant plasmid BD-OsIQMs_DelLQ_ and AD-OsCaM1 were transformed into the yeast AH109 strain, we can see all the transformants that were not able to grow on the SD/-Trp/-Leu/-Ade/-His medium (Figure 6B). The results suggested that the IQ motif is required for OsIQMs to combine with OsCaM1.

### 2.8. Expression Profiling of OsIQM Genes

**Figure 7:**
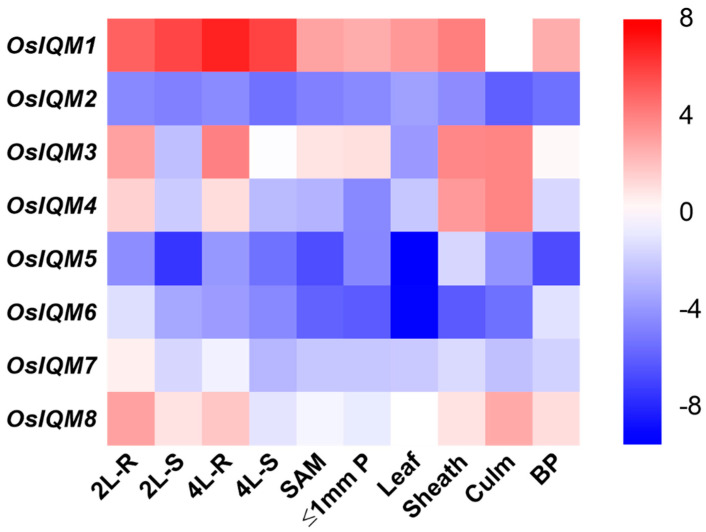
Heat map of the real-time quantitative PCR (qRT-PCR) analysis results of *OsIQMs* genes across different tissues analyzed, with three biological and technical replicates. The scale representing the relative signal intensity values is shown above. 2L-R—roots of 2-leaf stage seedlings; 2L-S—2-leaf stage seedlings; 10L-SA—shoot apex of 10-leaf stage seedling; ≤1P—developing panicles with a length of ≤1 cm; BP—booting panicle.

To examine the transcript of *OsIQM* genes in the entire rice life cycle, the expression profiles at different developmental stages and organs were analyzed by quantitative real-time PCR (Figure 7). RNA was extracted from various organs and tissues of Zhonghua 11 (ZH11). The expression patterns of the *OsIQM1* genes showed high expression in all tested organs and tissues. *OsIQM2, OsIQM5* and *OsIQM6* showed relatively low expression levels in all tested organs and tissues. *OsIQM3* and *OsIQM4* displayed higher expression in root, leaf sheaths and culm. *OsIQM7* was predominantly expressed in root, and *OsIQM8* showed high expression in root and culm.

**Figure 8:**
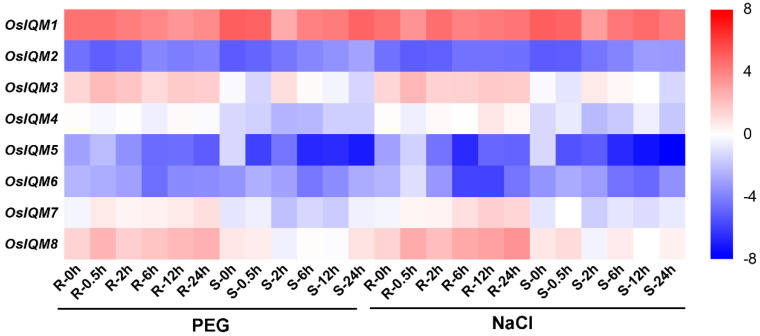
Heat map of the real-time quantitative PCR (qRT-PCR) analysis results of *OsIQMs* genes in shoots and roots under PEG and NaCl treatment, with three biological and technical replicates. The scale representing the relative signal intensity values is shown above.

In Arabidopsis, IQM1 was regulated by disease, ABA and JA [14,15]. However, no *IQM* genes responsive to stress and hormones have been reported in rice. We used qRT-PCR to analyze the expression levels of *OsIQM* family genes under stress and hormones treatments. As shown in Figure 8 and Figure S3, the *OsIQM* family have the same expression level under PEG and NaCl treatment. To realize the expression patterns of the eight *OsIQM* genes, we further revealed the expression levels of each gene for each period. Of the eight genes, four (*OsIQM3, 5, 7* and *8*) showed the higher transcript levels in the root treated with PEG, while three (*OsIQM2*, *3* and *5*) showed the higher transcript levels in the shoot under the PEG treatment. In the root, these genes caused a marked change in the transcription levels at 0.5 h.

**Figure 9:**
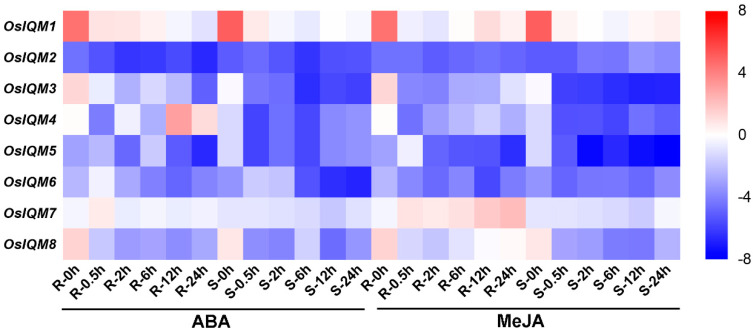
Heat map of the real-time quantitative PCR (qRT-PCR) analysis results of *OsIQMs* genes in leaves under ABA and MeJA treatment, with three biological and technical replicates. The scale representing the relative signal intensity values is shown above.

From the heat map of the qRT-PCR analysis results for the *OsIQM* genes, almost all genes were found to be ABA and MeJA responsive; however, some differences were observed among these genes (Figure 9 and Figure S4). Following ABA treatment, the transcript levels of three genes (*OsIQM1, 3* and *8*) were rapidly reduced in root and shoot, and *OsIQM4* was downregulated in the shoot but upregulated in the root. *OsIQM2* and *OsIQM7* genes exhibited minor changes in expression of shoot and root at all time points except *OsIQM7* was highly expressed at 0.5 h in the root. For *OsIQM5* and *OsIQM6*, the expression level was initially upregulated to high levels during early treatment, and then downregulated in both root and shoot. Under MeJA treatment, most of the *OsIQM* genes (*OsIQM1, 3, 4, 5, 6, 7* and *8* for the root, *OsIQM1, 2, 3, 4, 5, 6* and *8* for the shoot) were inducible by MeJA abiotic stress. Among these, the expression levels of *OsIQM1, 3, 4* and *8* were obviously downregulated in both root and shoot, while *OsIQM6* exhibited minor changes in downregulated expression. By contrast, *OsIQM5* was upregulated during early treatment which peaked at 0.5 h but downregulated at later time points for root, while *OsIQM7* was obviously upregulated at all time points. For the shoot, *OsIQM5* was obviously downregulated while *OsIQM2* was gradually upregulated. These results indicated that certain *OsIQM* genes play important roles in regulating the responses to PEG, NaCl, ABA and MeJA stress.

### 2.9. Co-Expression Network Analysis of OsIQMs

**Figure 10:**
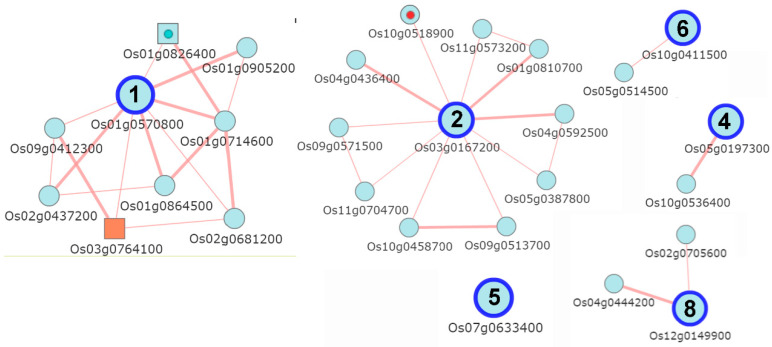
Co-expression network of the eight OsIQMs. The numbers represent the corresponding *OsIQMs* genes. The KEGG pathways are highlighted as dots: red—nucleotide excision repair (osa03420); blue—plant–pathogen interaction (osa04626).

To evaluate whether the genes co-expressed with *OsIQMs* involve in various biological processes, an extensive co-expression analysis of the eight *OsIQMs* was undertaken using the multiple guide gene search (MR = 10, hierarchy = 1) in the RiceFREND web servers [22]. The results indicated that *OsIQM1* was mainly involved in plant–pathogen interaction pathways by *WRKY24* (Figure 10). *OsIQM2* was involved in nucleotide excision repair pathways (Figure 10). No hits were found for *OsIQM3, OsIQM5* and *OsIQM7*.

## 3. Discussion

The plant-specific *IQM* gene family is widespread in many plants and are reported to participate in many crucial biological processes in plants [13,14,15,16]. The systematic and integrative analysis of *IQM* genes has been studied in Arabidopsis (Zhou et al., 2010). However, little is known about the rice *IQM* gene family. We focused on rice crop and performed genome-wide analysis to identify the *IQM* gene family in rice.

### 3.1. Evolution of the OsIQMs Gene Family in Rice

Genome-wide analysis of a gene family can characterize plant gene functions and facilitate the study of the evolution of genes and genomes. In the present study, based on the amino acid sequence of the conserved domain, eight *IQM* genes were identified in the rice genome. These were divided into three subfamilies based on their phylogenetic relationships with IQM proteins from *Brachypodium distachyon*, *Oryza sativa*, *Arabidopsis thaliana*, *Populus trichocarpa*, *Zea mays*, *Glycine max* and *Solanum lycopersicum* (Figure 1A). The phylogenetic analysis revealed the evolutionary relationships between the rice IQM proteins, and also showed that subfamily I contained the largest number (four) of rice IQM protein family members. Most closely related members within the same subfamily shared similarities in gene structure (Figure 2) and motif distribution (Figure 3), implying the reliability of the subfamily classification. Therefore, we speculated that the IQMs in each subfamily may have similar functions.

To further analyze the phylogenetic relationship of the *IQM* genes, we identified the putative orthologous and paralogous genes from members of our phylogenetic tree. Generally, orthologs are defined as genes from different genomes that were derived from a single ancestral gene and may have similar functions. In our study, the putative orthologous genes of *OsIQMs* (*OsIQM1/Bd2g41490*, *OsIQM4/Bd2g33150, OsIQM5/Zm2g318843, OsIQM6/Zm2g168222* and *OsIQM8/Bd4g42560*) were identified (Figure 1A). Notably, the ortholog pair formed by eudicot and monocot was not found in the phylogenetic tree, indicating before the eudicot–monocot split, there were no ancestral *IQM* genes that existed.

In addition, paralogs are defined as genes that originate from an ancestor gene within the same genome produced by gene duplication events [23,24]. Therefore, gene duplication events play an important role in the evolution of a gene family [25,26]. In our study, we found that all *OsIQM* gene pairs exhibited segmental duplication events but not tandem duplication events (Figure 4A). This result indicated that segmental duplication played a leading role in the expansion of the rice *IQM* gene family. To better explain the patterns of expansion, estimates of the evolutionary rates are extremely useful. We estimated the Ks and Ka models of paralogous genes and calculated the approximate date of the duplication event. By calculating the duplication dates for the paralogous pairs, we concluded that all of the segmental duplication events in the *OsIQM* gene family occurred ranged from 11.9 to 19.8 Mya. Therefore, before the evolutionary expansion of the *OsIQM* gene family in the rice genome, five *OsIQM* genes were distributed on four chromosomes. From the separation of *Zizaniinae* and *Oryzinae* (approximately 20 million years ago) to the time where *Oryza* branched-off from the remaining *Oryzeae* genera (approximately 9 million years ago), *OsIQM5* and *OsIQM6* arose from *OsIQM2* and *OsIQM3* on chromosome 3, *OsIQM8* arose from *OsIQM7* on chromosome 11 through segmental duplication event. After the duplication evolutionary stages, the number of *OsIQM* gene family increased to eight genes.

### 3.2. The IQ Motif Plays an Important Role in the Interaction of OsIQM and OsCaM

Previous studies have revealed that five classes of IQ motif-containing protein family (CNGC, IQM, CAMTA, myosin and IQD) had divergence in the number and distribution of IQ motifs [11]. The IQ domain was related to the calmodulin-binding domain, and it was reported that IQD proteins interact with CaMs in maize [27], Chinese cabbage [28], moso bamboo [29] and Arabidopsis [30], IQM proteins interact with CaMs in Arabidopsis [13]. In our study, multiple sequence alignments of the amino acid sequences of eight OsIQM proteins revealed the presence of the IQ domain (Figure S1). Moreover, MEME analysis showed that motif one, motif two, motif three, motif four, motif five and motif six were present in almost all members of the OsIQM family and motif three contained the IQ motif. Next, we used yeast two-hybrid analysis to detect the interaction between OsCaM and OsIQMs. The results showed that OsIQMs interacted with OsCaM, which provide another evidence that OsIQMs protein might function throughout the CaM pathway. Previous studies have shown that AtIQM1 can bind with CaM5 via its IQ-motif. In order to further understand whether the IQ motif plays an important role in the interaction of OsIQM and OsCaM, we deleted the LQ of OsIQM to test the binding activity of OsIQM and OsCaM. The results indicated that the IQ motif is the key domain of OsIQM and determined the binding activity of OsIQM and OsCaM. These preliminary results of the *OsIQM* family genes may provide a foundation for further studies on the biochemical functions of IQM proteins in rice.

### 3.3. OsIQMs Respond to Abiotic and Biotic Stress and May Take Part in Stress Resistance Involving Stress-Related Genes

The evolutionary expansion of a gene family may result in functional diversification by gene duplication, and then perform the different expression profile of the gene family member [25,31]. To further investigate the possible functions of the OsIQMs in plant growth and development, the spatio-temporal expression patterns of the eight *OsIQMs* in ten different tissues and organs were detected by qRT-PCR (Figure 7). Based on our results, the *OsIQM* genes were differentially expressed at various developmental stages and tissues. *OsIQM1* exhibit high expression levels in all tissues. By contrast, *OsIQM2, OsIQM5* and *OsIQM6* lowly expressed across all tissues, and indicated that these genes may work together with other proteins during plant growth and development. In addition, unlike most duplicated genes, the expression profiles of the duplicated *OsIQM* genes had distinct tissue specificities.

Jasmonic acid (JA) is an essential plant hormone that regulate plant growth and developmental processes as well as disease response signaling [32]. The catalases are involved in plant stress resistance by eliminating excess H_2_O_2_ in Arabidopsis and rice [33,34]. In Arabidopsis, IQM1 can positively regulate JA content and *B. cinerea* resistance by directly interacted with and promoted CATALASE2 (CAT2) expression and CAT2 enzyme activity, indicating that IQM1 is a key regulatory factor in the signaling of plant disease responses mediated by JA [14]. Therefore, we used qRT-PCR to investigate the expression patterns of *OsIQMs* family genes under MeJA treatments (Figure 9). The results demonstrated that most *OsIQMs* genes were either increased or repressed under the MeJA treatments, and we speculated that *OsIQMs* genes might have a similar biological function in the signaling of plant disease responses. It has been reported that WRKY transcription factors played an important role in abiotic stress responses [35,36] and biotic stress responses [37,38,39,40]. OsWRKY11, 24, 30 and 71 are transcriptional activators that positively regulates disease resistance in rice [35,41,42,43]. In this work, co-expression networks showed that seven *OsIQM1* co-expressed with *OsWRKY24*, implying that OsIQM1 might partner with OsWRKY24 during plant disease response (Figure 10). Meanwhile, the results from the Genevestigator demonstrated that the expression of *OsIQM1* was rapidly increased after infection of *Magnaporthe oryzae* (*M. oryzae)* (Figure S5)*. These results further demonstrated that* OsIQM1 may be involved in disease resistance in rice, similar to AtIQM1. In addition, the phytohormone, ABA, plays a predominant role in regulating plant adaption to various abiotic stresses such as drought, salt or low temperature [44]. Zhou et al. verify that *AtIQM4* plays key roles in modulating the responses to ABA, salt and osmotic stress during seed germination and post-germination growth [15]. Similarly, the expression of most *OsIQMs* members was regulated by ABA, PEG, NaCl. Thus, it is possible that these *OsIQMs* respond to abiotic and biotic stress and may be candidate genes for stress resistance in rice. The new information obtained in this study may provide new insights for further functional characterization of *OsIQMs*.

## 4. Materials and Methods

### 4.1. Identification of IQM Genes in Rice

The current study searched rice IQM (OsIQM) proteins using the Basic Local Alignment Search Tool algorithms program (BLASTP) in RAP-DB (http://rapdb.dna.affrc.go.jp/, accessed on 19 July 2020) and NCBI database (http://www.ncbi.nlm.nih.gov/, accessed on 19 July 2020), with the Arabidopsis IQM protein sequences used as query sequences. After manually removing the redundancy genes based on cluster-W alignment results, all candidate sequences were further confirmed using SMART (http://smart.embl-heidelberg.de/, accessed on 19 July 2020) for detecting the IQ domain [45]. Sequences of *Arabidopsis thaliana* IQM proteins were retrieved from the Arabidopsis Information Resource (TAIR, http://www.arabidopsis.org/, accessed on 19 July 2020), *Brachypodium distachyon*, *Populus trichocarpa*, *Zea mays* and *Glycine max* IQM protein sequences were downloaded from Phytozome v12.1, (http://www.phytozome.net/, accessed on 19 July 2020) and *Solanum lycopersicum* IQM protein sequences were retrieved from the tomato WGS chromosomes (2.40; SL2.40) (SGN http://solgenomics.net, accessed on 19 July 2020), respectively. Information on rice *IQM* genes, including the number of amino acids, open reading frame (ORF) lengths and location coordinates were acquired from RAP-DB. The physicochemical parameters of each rice proteins were generated by the program ExPASy (http://web.expasy.org/protparam/, accessed on 11 August 2020). The subcellular localization of the IQM proteins were predicted with the WoLF PSORT program (http://wolfpsort.org, accessed on 11 August 2020) using the amino acid sequences.

### 4.2. Chromosomal Localization and Gene Duplication

The chromosomal distribution of *OsIQM* genes was drafted by MapInspect software (http://www.plantbreeding.wur.nl/uk/software_mapinspect.html, accessed on 15 August 2020) according to their position information available in the Rice Genome Annotation Project (RGAP) database (http://rice.plantbiology.msu.edu/index.shtml, accessed on 15 August 2020). The current study analyzed the *OsIQM* gene duplication events, including tandem and segmental duplications. If the *OsIQM* gene were placed on duplicated chromosomal blocks available at MSU-RGA with the maximal length distance permitted between collinear gene pairs of 500 kb, they were produced by segmentally duplicated events. Tandem duplicates were determined when genes separated by five or fewer genes in a 100-kb region.

To further analyze the divergence of duplicated genes, the synonymous substitution rate (Ks) and non-synonymous substitution rate (Ka) were calculated using the software DnaSp. Based on a rate of λ (λ = 6.5 × 10^−9^ for rice) substitutions per synonymous site per year, the divergence time (T) was subsequently calculated using the equation T = Ks/2λ × 10^−6^ million years ago (Mya).

### 4.3. Protein Sequence Alignment and Phylogenetic Analysis

Multiple sequence alignment of all IQM protein and conserved domains (IQ domain) was performed using the Clustal X (version 1.83) program. To generate the unrooted phylogenetic tree, the neighbor-joining (NJ) method was used and with the *p*-distance substitution model in the MEGA X software. For bootstrap analysis we used 1000 replicates and the pairwise deletion option to obtain a support value for each branch.

### 4.4. Analysis of Gene and Protein Structure

For gene structure analysis, the coding sequence (CDS) and corresponding genomic sequences of *OsIQM* genes were downloaded using genome browser tool in RAP-DB, and we used the Gene Structures Display Server2.0 (GSDS2.0) (http://gsds.cbi.pku.edu.cn/, accessed on 1 September 2020) to analyze the exon/intron structure for individual *IQM* genes.

To investigate the conserved motifs of IQM proteins, the complete amino acid sequences were analyzed using MEME (Multiple Expectation Maximization for Motif Elicitation) (http://meme.nbcr.net/meme/cgibin/meme.cgi, accessed on 1 September 2020). The maximum number of motifs were defined as 10, the motif width was set to between 6 and 200 residues, respectively.

### 4.5. Yeast Two Hybrid Assay

The Matchmaker GAL4 two-hybrid system was used to test the interaction between IQMs and CaM. For yeast two-hybrid assay, the full-length CDS of *CaM* were separately cloned into pGADT7, the full length of three *IQM* CDSs was cloned into the pGBKT7 bait vector. They were co-transformed into AH109 yeast cells by the PEG/LiAC method. Interactions in yeast were determined on SD/-His-Trp-Leu-Ade plates. The co-transformants with pGBKT7-53 and pGADT7-T were used as positive control while pGBKT7-Lam and pGADT7-T were used as negative controls.

### 4.6. Effects of Mutations in IQ Motif of OsIQMs on Its CaM Binding

To confirm the effects of mutation of key amino acid residues in the IQ motif of IQM on its CaM binding, the LQ was deleted through the mutagenesis technology in vitro. The PCR products were subsequently treated with Dpn I (TaKaRa, Japan) to eliminate the methylation. The OsIQMs_DelLQ_ fragment (OsIQM1_Del140-141VQ_, OsIQM2_Del125-126VQ_, OsIQM3_Del112-113LQ_, OsIQM4_Del11-112VQ_, OsIQM5_Del103-104LQ_, OsIQM6_Del140-141LQ_, OsIQM7_Del43-44LQ_, OsIQM1_Del41-42LQ_) was inserted into the pGBKT7 vector. Then CaM binding of the mutant proteins was analyzed via yeast two-hybrid assay described above.

### 4.7. Plant Materials and Growth Conditions

Rice (*Oryza sativa* L. ssp. *japonica* cv. Zhonghua 11) seedlings were used as the WT control in this study. In order to evaluate the spatio-temporal expression profiles of *OsIQM* genes under normal conditions, the rice seedlings were grown in a controlled paddy at the South China Botanical Garden during natural growing seasons (March to July). For various treatments, seeds were surface-sterilized with ethanol (70% *v*/*v*) for 1 min and then with 5% NaOCl solution for 50 min, followed by several rinses with sterile water and cultured in Hoagland’s Solution at 28 °C under 14 h light and 10 h dark for 2 weeks. For analysis of gene expression under abiotic stresses, 2-week-old ZH11 seedlings were exposed to the following treatments: salinity (150 mM NaCl) and PEG (20% PEG6000) [46]. For analysis of gene expression with hormone, ABA (50 μM) and MeJA (50 μM) were added. Shoot and root were sampled at 0, 0.5, 2, 6, 12 and 24 h after treatment, then immediately frozen in liquid nitrogen and stored at −80 °C [46].

### 4.8. RNA Isolation and Quantitative Real-Time PCR

The total RNA was extracted from different rice samples according to the manufacturer’s protocols of RNAiso Plus (Takara, Code No. 9108). M-MLV Reverse Transcriptase (Promega, Cat#M1701) was used for reverse transcription. qRT-PCR was performed on 384-well plates with SYBR Premix Ex Taq II (Takara, RR820A) using a Roche Light Cycler 480 Real-Time PCR system. *eEF-1a* were used as internal controls for mRNA and the relative expression levels of genes were calculated using the 2^−^^△△CT^ method. The experiments were performed in triplicate. All primers used are listed in Table S1.

### 4.9. Subcellular Localization Assays

A green fluorescent protein (GFP) fusion protein was constructed using full-length *IQM* CDS with a C-terminal fusion of the GFP clone under the control of a CaMV35S promoter [47]. Arabidopsis protoplast isolation and transformation were conducted as previously described [48]. The protoplasts were incubated at 22 °C for 12 h in the dark after transfection, and the subcellular distribution of the GFP fusion protein was examined using a confocal laser scanning microscope. Excitation was achieved using an argon laser at 488 nm (GFP), and the emission of GFP was detected from 492 to 550 nm. The auto-fluorescence of chlorophyll was simultaneously detected between 650 and 730 nm. The images presented are average projections of 8–20 optical sections [47].

### 4.10. Co-Expression Network Analysis

The *OsIQMs* was employed as a guide gene in Rice FREND (http://ricefrend.dna.affrc.go.jp/, accessed on 1 September 2020) [22]. In Rice FREND, Mutual Rank (MR) values were assessed between *OsIQMs* and co-expressed genes.

## 5. Conclusions

The current study systematically identified and characterized the plant-specific *IQM* gene family in rice. In this study, eight *IQM* genes identified in the rice genome were classified into three subfamilies (I–III). Gene structure and protein motif analyses showed that these genes are relatively conserved within each subfamily. Furthermore, quantitative real-time PCR analysis of eight *OsIQMs* genes showed that most *IQM* genes were responsive to PEG, NaCl, MeJA and ABA treatment, suggesting their crucial roles in biotic and abiotic stress response. Additionally, yeast two-hybrid assay showed that OsIQMs can interact with OsCaMs, and the IQ motif of OsIQMs is required for OsIQMs to combine with OsCaMs. Our preliminary results may be useful for future elucidating the role of IQMs in rice, bringing us one step closer to understanding the important biological functions of these proteins.

## Figures and Tables

**Table 1:** Eight identified members of the rice *IQM* gene family.

Locus ID	Gene Name	Chr ^a^	Pos ^b^	ORF Length (bp)	Number of Amino Acid	Molecular Weight (Mw/Da)	Theoretical pI	FL-cDNAc	WoLF PSORT ^c^
Os01g0570800	*OsIQM1*	1	21900067	1710	570	63,264.22	9.15	AB190923	CH
Os03g0167200	*OsIQM2*	3	3631183	1833	611	68,581.08	9.28	AK066554	N
Os03g0374500	*OsIQM3*	3	14739099	1734	578	63,230.78	6.43	AK071310	N
Os05g0197300	*OsIQM4*	5	5996776	1671	557	61,573.53	8.89	AK106389	N
Os07g0633400	*OsIOM5*	7	26287974	1758	586	65,197.82	6.19	AK071894	N
Os10g0411500	*OsIQM6*	10	14319443	1950	650	73,057.63	8.25	AK072572	N
Os11g0151002	*OsIQM7*	11	2367903	1425	475	52,247.14	9.15	AK102404	N
Os12g0149900	*OsIQM8*	12	2451806	1434	477	52,997.92	8.37	AB164644	N

^a^—the chromosome in which the gene is located. ^b^—the start position of the gene on the chromosome. ^c^—CH chloroplasts, N nucleus.

## Data Availability

Not applicable.

## Supplementary Materials

The following are available online at https://www.mdpi.com/article/10.3390/plants10091949/s1, Figure S1: amino acid sequence alignments of IQ domains in rice IQM protein sequences. The multiple alignment results indicate the highly conserved IQM domains among the eight identified rice IQM protein sequences. The IQ motif was in the red box. Figure S2: the conserved amino acid sequences and length of each motif. Figure S3: expression patterns of eight OsIQM genes under PEG and NaCl stress, as revealed by qRT-PCR. Figure S4: expression patterns of eight OsIQM genes under MeJA and ABA stress, as revealed by qRT-PCR. Figure S5: the expression of OsIQM1 was rapidly increased after infection of M. oryzae from the Genevestigator. Table S1: all primes in this study.
