# Supplementary material for: Genome-Wide Analysis of the IQM Gene Family in Rice (Oryza sativa L.)
Source: Plants (Basel). 2021 Sep 18;10(9):1949. doi: 10.3390/plants10091949 (PMC8469326; doi:10.3390/plants10091949)
Supplement: Supplementary file 1 [file plants-10-01949-s001.zip › plants-1349193-supplementary.pdf]

```
OsIQM2    1 MGLSISYPPDDYLPTEED-TDQMFVRSLSSDNLSTIETFESP---------PALLDSLSSQ-RPIIKESFNFKKSEGDPFHVETTVSLMS
OsIQM6    1 MGLSISYPPDDYLPAMEDNMGRLFIRSLSFDDDMEAAADDSPSTSPMPSPSATLLPAFGSGGKLIIEGSLSFKRREADPVQMETMISIRS
OsIQM3    1 MGVLFSCPVDDYDALEES------AAAAAAASSESNSGGGKP---------AAILKALGSG-KLLIEGSLSFKRDQQMSPTSLLQVETEI
OsIQM5    1 MGVLFSCPVDDETAAVED------AAPVAEQ--------------------AVLKASLGSGGRLRIEGSLSFKTREQQSLQVETKIPAVT
OsIQM7    1 MAVATAAAGLDHAGSSR--------------------------------------------------------------LDAEVSSPVAG
OsIQM8    1 MGVDAAPDRMDAASSWMN-------------------------------------------------------------PPVEVSSPVAG
OsIQM1    1 MTLRPLNTERSFLLSSPKPHSPRDACSPPVRSPSSTRLLACRKLPSSSKPMATGAGVLERSLSFKNWEPTAAEEAAVAAPPPHDEAASRC
OsIQM4    1 MGLH----QHAWGEVGIEIFSPRSKHIAMVNKISPWDQQEKMALRSN--------GALVKSLSFKEWEGGEQTKTNSVNHKNRPSLINVV

OsIQM2   80 PKPGKEKSCTHKRTILPRYG----SMEYLPPHSPVVG-MISPKHQAAAVRVQKVYKSFRTRRQLADCAVLVEQRWWKLIDFALLKHNSVS
OsIQM6   91 PKSDKE-SCSSKPNATAGASRFALAGDQTPEDSPVIAGVASPKHQAAAVRLQKVYKSFRTRRQLADCAVLVEQSWWKLLDFALLKRNSVS
OsIQM3   75 S---IKPAAADIA-AAPRAR---------FAADGGAAAAESPKHEAAAVKLQKVYKSFRTRRQLADCAVLVEQSWWKLLDFALLKRSSVS
OsIQM5   65 S---PRAAPAPMPRELLRTR---------FADAAAAAAPESPKHEAAAVTLQKVYKSFRTRRRLADCAVLVEQSWWKLLDFALLKRSSVS
OsIQM7   29 S------------------------------------GGGGGGEDGAATKLQKMYRSYRTRRKLADTAVVVEELWWQALDYARLSYSTIS
OsIQM8   30 ---------------------------------------GGGGEDGAATKLQKVYRSYRTRRKLADSAVVVEELWWQALDFARLSHSTVS
OsIQM1   91 INGAR-PGILLLQQSPKAKQGDAATSPAQAALIEFISPKPRSELDQAATKVQKLFKGHRTRRNLADCAIVVEELWWKAYDSACLNIKSIS
OsIQM4   79 VDNRRNSDIFMAESSP------------------IVSSSPKCELDAAAVKVQKVYKSYRTRRNLADCAVVVEELWWKALDFASLKHSSIS

OsIQM2  165 FFEEEKPESALSRWSRARTKAAKVGKGLSKDEKAQKLALQHWLEAIDPRHRYGHNLHYYYQHWLHCESKQPFFYWLDVGEGKDVSMEDHC
OsIQM6  180 FFDIEKPETAISRWSRARMRAAKVGKGLSKDEKAQKLALQHWLEAIDPRHRYGHNLHYYYHRWLHCEINQPFFYWLDVGEGKDVNLEEHC
OsIQM3  152 FFDIEKQETAVSRWSRARTRAAKVGKGLSKDEKAQKLALQHWLEAIDPRHRYGHNLHYYYDTWLHCESKQPFFYWLDVGEGKEINLEGKC
OsIQM5  143 FFDIEKQESAVSKWARARTRAAKVGKGLSKDDKAQKLALQHWLEAIDPRHRYGHNLHYYYDCWLQCESKEPFFYWLDVGEGKEINLEDRC
OsIQM7   83 FHD-PNPETVASRWSRVSIIASKVGQGLSRDAKARKLAFQHWIEAIDPRHRYGHNLQCYYDVWCQSQAGQPFFYWLDIGEGKDVDLP-EC
OsIQM8   81 FFDDPKPETVASRWNRVSLNASKVGQGLSRDGKALKLAFQHWIEAIDPRHRYGHNLHFYYDVWCQSQAGQPFFYWLDIGEGKDVDLP-EC
OsIQM1  180 FFDEAKQETAASRWSRAGKRIAKVGKGLSKNEKAQKLALQHWLEAIDPRHRYGHNLHLYYDIWSASSSTEPFFYWLDVGAGRDMHHQ-KC
OsIQM4  151 FFNGEKPETAASRWARARTRAAKVGKGLSKNGKAQKLALQHWLEAIDPRHRYGHNLHIYYGVWSRSESTEPFFYWLDIGEGKEVNLD-RC

OsIQM2  255 PRWKLLQQCIRYLGPKEREFYEVVIENKKLLYKMSRKIVDTSEGPKNSKWIFVLSTTRVLYIGTKSKGTFQHSSFLAGGATSAAGRLVVD
OsIQM6  270 PRWKLHKQCIKYLGPKERESYEVIVEDSRLIYKLSRQIVNTTKSRKGSKWIFVLSTCKTLYIGQKQKGTFQHSSFLAGGATSAAGRLIVE
OsIQM3  242 SRSKLLSQCIKYLGPKEREDYEVILEDGKFLYKKSRQILDTSCGPRDAKWIFVLSTSKSLYVGQKKKGKFQHSSFLAGGATSAAGRLVVE
OsIQM5  233 PRWKLLSQCIKYLGPQEREDYEVVIEDGKFMYKNSREILDTSGGPRDDKWIFVLSTSKNLYVGQKKKGKFQHSSFLAGGATSAAGRLVVE
OsIQM7  171 PRAQLKKQCIKYLGPQEREQYEYIITKGKIIHKYSEEPLDTSQG---SKWIFVMSTTKRLYAGKKEKGVFQHSSFLAGGATIAAGRFTAE
OsIQM8  170 PRARLKKQCIKYLGPQERELYEYIVTEGKIIHKHSGEPLDTSQGPKGTKWIFVMSTTKKLYAGKKERGVFQHSSFLAGGATIAAGRFTAE
OsIQM1  269 PRSKLYSQLIMYLGPNEREAFEVVVEGGKLMYRKSGVLVNTTED---SKWIFVLSTTRSLYVGQKKKGKFQHSSFLAGAATTAAGRLVAK
OsIQM4  240 PRNKLQSQCVKYLGPKERQEYEVVVESGRLVYKQSGVFVHTSDD---SKWIFVLSTTKALYVGQKKKGSFQHSSFLAGGAITSAGRLVVK

OsIQM2  345 NGILKAVWPHSGHYRPTEANFREFMMYLKKRNVDLANIKLSPSEDEEDECLRSRSGRSQ---LEPTEPGKPEKEEDATADDNGTTTVAAQ
OsIQM6  360 DGILKAVWPHSGHYRPTEQNFQEFMNFLKERNVDLTDVMLNPSEGEDDAEFSLKSSHSRQDLTELCEPDMQEHEEQVTQHHGADETKTSS
OsIQM3  332 NGTLKAIWPHSGHYRPTEENFEEFKSFLNDNSVDLTDVKMSPAEEDEEFWGSLKRISSES-YPKNTATDNSEDQAAEAEETGNSQMPRVS
OsIQM5  323 DGTLKAIWPHSGHYRPTEENFQEFQGFLKDNNVDLTDVKMSPTEEDEEFWSRLRSIPSDR-CAD--AADNTEEEMNSSEQTVNCQTPEAT
OsIQM7  258 NGVIKSIWAYSGHYKPSAENLSNFMNFLEENGVDLN----------NVYEEP--------------VPNKVQSPITAIIESNPPQLILPQ
OsIQM8  260 NGVIKSIWAYSGHYKPSAENLANFMNFLEENGVDLKEVEVRSSTNEDYYEDP--------------VPNK-QNPLATVMESNPPQLILPQ
OsIQM1  356 DGVLQAIWPYSGHYLPTEENFREFISFLEENSVDLADVKRCSVD-DDEFPSF----------------KKTEEKPE--------------
OsIQM4  327 DGILKAIWPYSGHYLPTEENFREFISYLQENGVDLADVKRCPMDKDDEYPLL----------------TKPDVTAASIAATKNVEKVAAA

OsIQM2  432 AAPPST-TGGEPATPVMKRSSSGNRLQRKRPPRLTLD-------------KSRLAKGVAEQDAGSFGDRLDFCKVNLFRG-GEEAEE---
OsIQM6  450 DAPTMTSTETMASTPAIRKSTSANKLQGKRPPRLLISSNNTELPATHCNGRPSPVHKDIDEDSTMFGECLAFCKKNLFAEEGNEEDE---
OsIQM3  421 DEP----TCAEIDG-CDEPAATR-RVDSS----------------------------SAVAAAAENTEAEE--ED---QEGGGE------
OsIQM5  410 ETPTEEISSQHIQETINNPSTTLPRVASSE--------------------------GPATSNAGDNGSSEEGGEDHHRQEEGDEPSSPSS
OsIQM7  324 NMVLENKASGSSSQVEGAEGDNAATEQAKP--------------------------TYQRTLSGGLQSPRAT------------------
OsIQM8  335 NMIEEDKASEPFSQAEGAESDNVPKVQTKP--------------------------TYQRTLSGGLKSPRA-------------------
OsIQM1  415 ---EAEKPTEPTHDEIMDSSQ------------------------------------------IELPEVDI-------------------
OsIQM4  401 TAAAAERLTETVSDDTDHAAVDEDGSMSEG-----------------------------EDEDADVPTATK-------------------

OsIQM2  504 AVVVPQEKILRRLNSRMTMNSYQLGKQLSLRWTTGAGPRIGCVRDYPPELQFRVMEQISLTPRGGAG-----PLRLGSTATPRQSPCAPL
OsIQM6  537 LVEVPEEMIMNRINCKKATKSYQLGKQLSFQWSTGAGPRIGCVRDYPSELQFRALEEVSLSPRG---------TRSTRFSSPRRKPLTPN
OsIQM3  466 QAPVPREKILERINSKKGMRSYQLGKQLSFRWTTGAGPRIGCVRDYPSELQLRALEQVNLSPRS--A-----AAAS---ASSRFSSPQRR
OsIQM5  474 SSSVPREKILERINSKKEAKSYQLGKQLSFKWTTGAGPRIVCVRDYPSELQLRALEQVHLSPRS--A-----AAAAGGRPSSRFASPQRS
OsIQM7  370 -IDVPRKAILERVKSKRESRSYQLGHKLSLKWSTGAGPRIGCVKDYPMQLRMQALEMVNLSPRA-------------SAPSISRRLQASL
OsIQM8  380 -ADVPREAILERVKSKGESKSYQLGHRLSLKWSTGVGPRIGCVKDYPMELRMQALEMVNLSPRA-------------STPSPSWRLPACL
OsIQM1  441 ---VKEAVVENS---EDTKVAPIMASRPSFKWATANGARIGCVRDYPADLQSMALEHVNLSPRVVPS----PTTNRLPIPSPRPSPKIRL
OsIQM4  443 ---EDEHKATSSSAASTTAAAAAAENHLPCRWSTGTGPRIRCVRDYPQDLQSRALEHVNLSPRLAAAGVAPATRKRDPVPSPRPSPGMIL

OsIQM2  589 PSPAPLYAAAGTPTS------LLQHGAA---------------------
OsIQM6  618 SIPVARFGCSPTAQGDNMGLKPRQRCATWTAF-----------------
OsIQM3  546 S---FNGAAAPATPREALRPSPLQHGLVATVAAAD--------------
OsIQM5  557 SSPMARGCSEPLTPREAFR-SHLQQGVLIR-------------------
OsIQM7  446 SLSPNLPTPPEFTTTQMAAPTKLEPATSI--------------------
OsIQM8  456 SPTPNLPSSP-LGPIQTSLP---QPS-----------------------
OsIQM1  521 SPRLHYMGLPTPTGCKLPIPSPEIRRSPRDQFMGFQTPSVSLTLPKLGK
OsIQM4  530 SPRLASVGFRPPV---VALTLPDFKRS-RLQ------------------
```

Figure S1. amino acid sequence alignments of IQ domains in rice IQM protein sequences. The multiple alignment results indicate the highly conserved IQM domains among the eight identified rice IQM protein sequences. The IQ motif was in the red box.

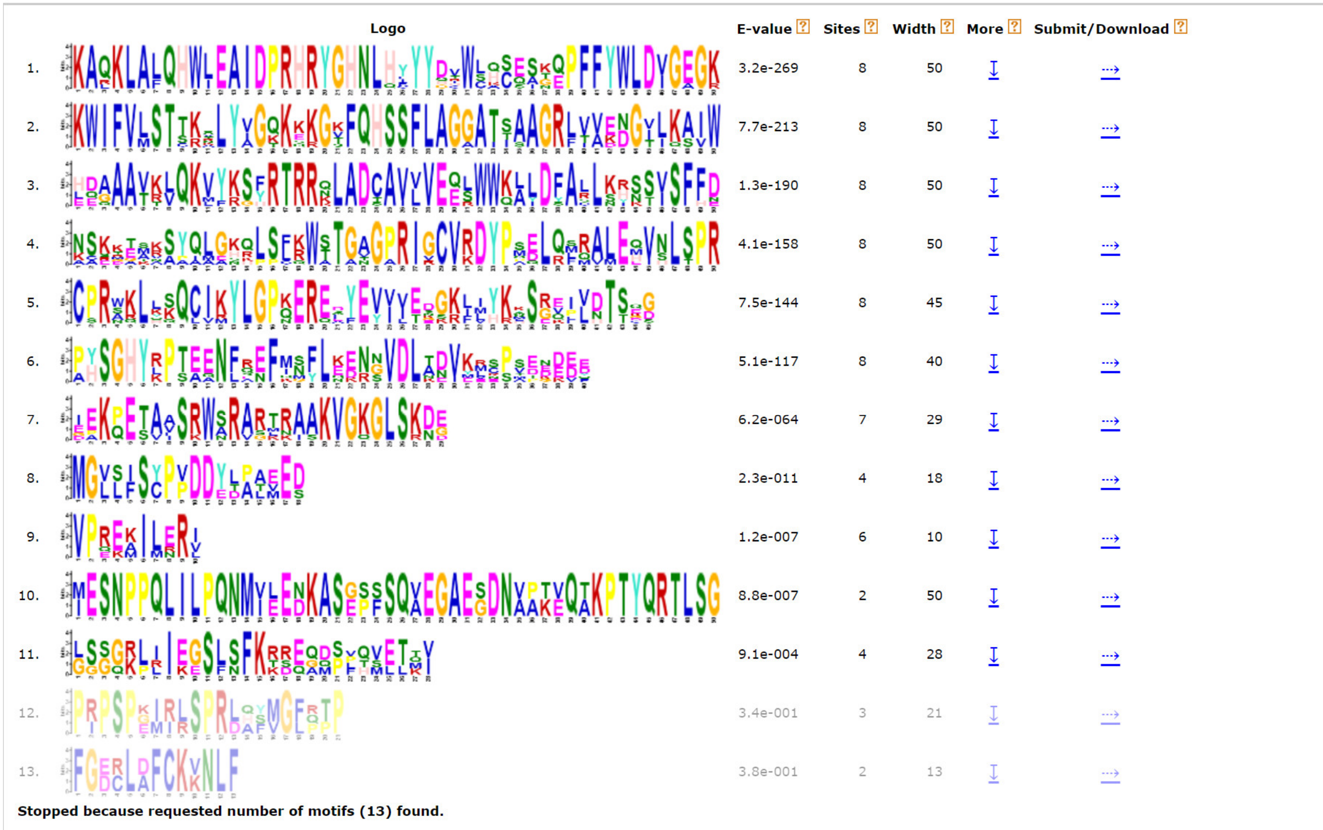

Figure S2: the conserved amino acid sequences and length of each motif.

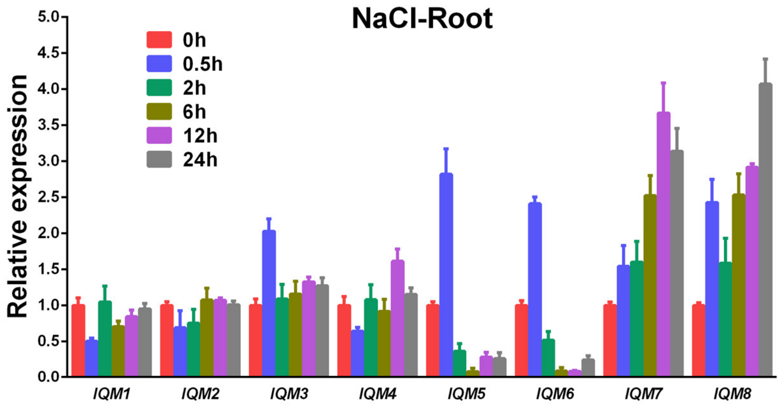

NaCl-Root
Relative expression
0h
0.5h
2h
6h
12h
24h
IQM1
IQM2
IQM3
IQM4
IQM5
IQM6
IQM7
IQM8

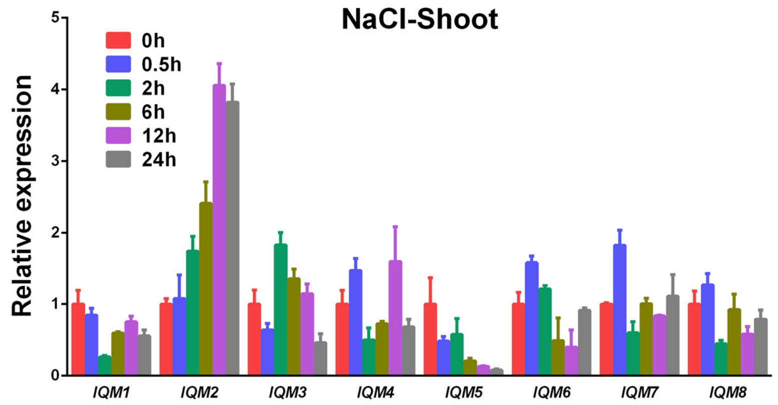

NaCl-Shoot
Relative expression
0h
0.5h
2h
6h
12h
24h
IQM1
IQM2
IQM3
IQM4
IQM5
IQM6
IQM7
IQM8

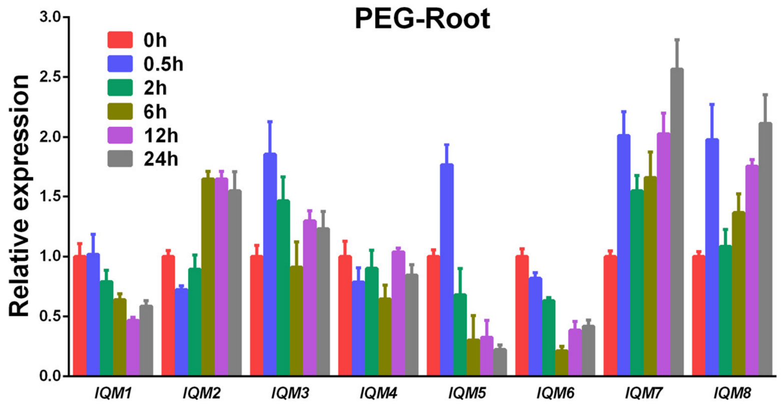

PEG-Root
Relative expression
0h
0.5h
2h
6h
12h
24h
IQM1
IQM2
IQM3
IQM4
IQM5
IQM6
IQM7
IQM8

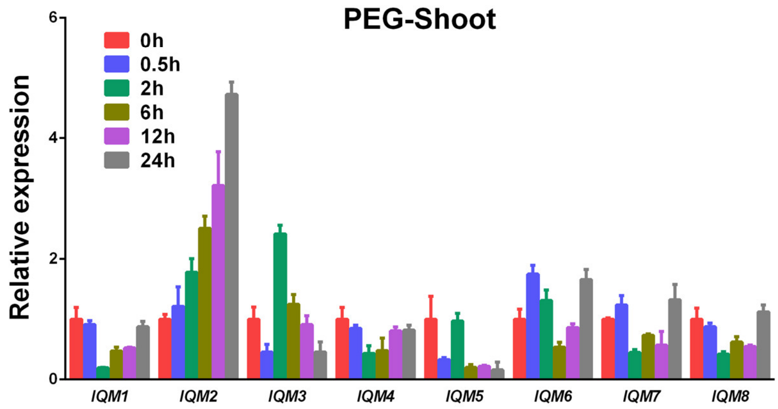

PEG-Shoot
Relative expression
0h
0.5h
2h
6h
12h
24h
IQM1
IQM2
IQM3
IQM4
IQM5
IQM6
IQM7
IQM8

Figure S3: expression patterns of eight OsIQM genes under PEG and NaCl stress, as revealed by qRT-PCR.

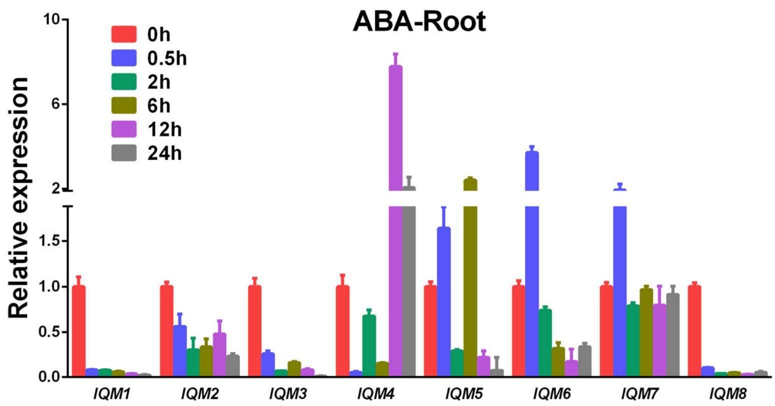
ABA-Root
Relative expression
0h
0.5h
2h
6h
12h
24h
10
6
2
1.5
1.0
0.5
0.0
IQM1
IQM2
IQM3
IQM4
IQM5
IQM6
IQM7
IQM8

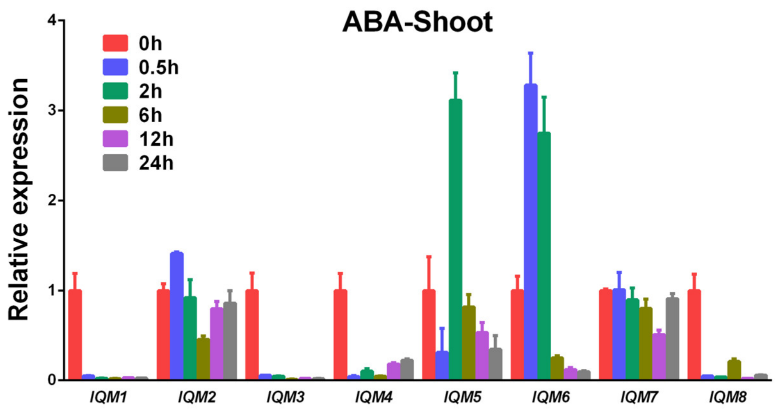
ABA-Shoot
Relative expression
0h
0.5h
2h
6h
12h
24h
4
3
2
1
0
IQM1
IQM2
IQM3
IQM4
IQM5
IQM6
IQM7
IQM8

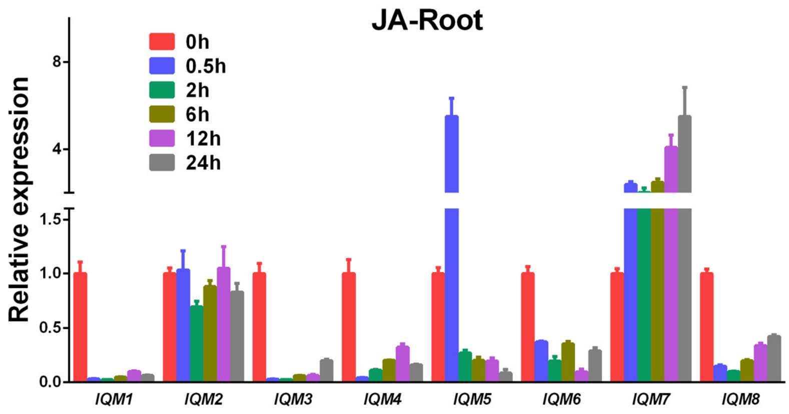
JA-Root
Relative expression
0h
0.5h
2h
6h
12h
24h
8
4
1.5
1.0
0.5
0.0
IQM1
IQM2
IQM3
IQM4
IQM5
IQM6
IQM7
IQM8

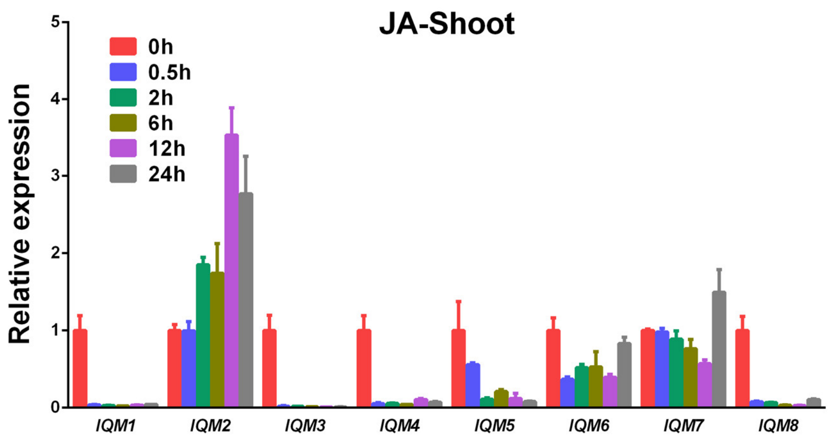
JA-Shoot
Relative expression
0h
0.5h
2h
6h
12h
24h
5
4
3
2
1
0
IQM1
IQM2
IQM3
IQM4
IQM5
IQM6
IQM7
IQM8

Figure S4: expression patterns of eight OsIQM genes under MeJA and ABA stress, as revealed by qRT-PCR.

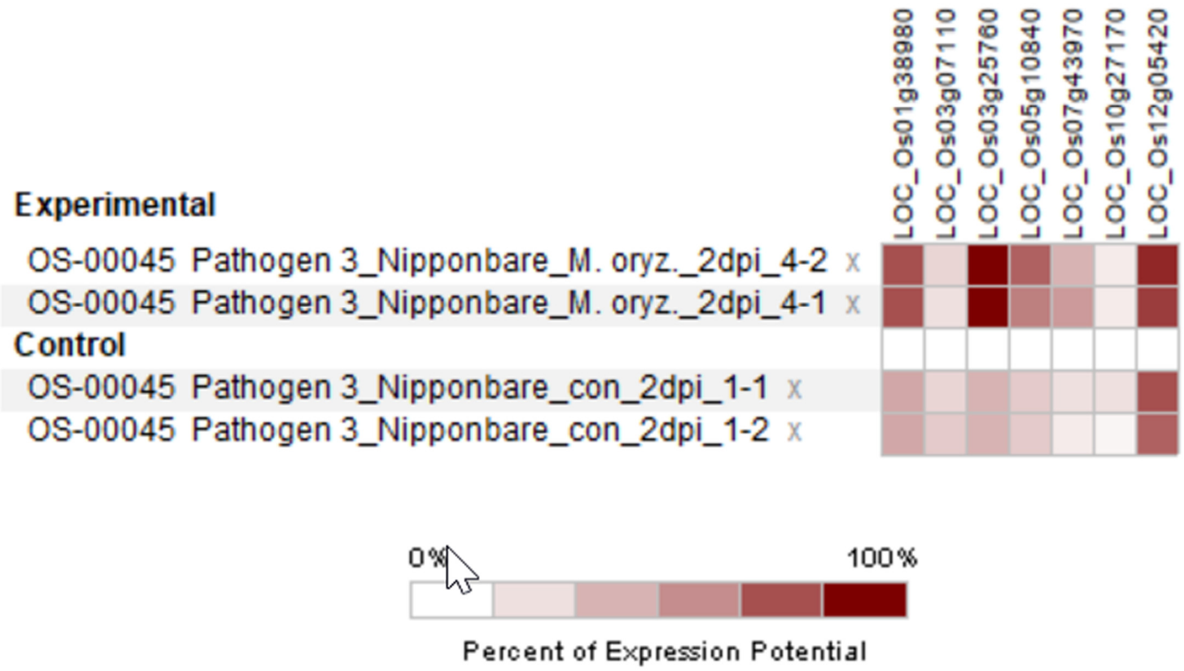

Figure S5: the expression of OsIQM1 was rapidly increased after infection of M. oryzae from the Genevestigator.

Table S1. List of the primers in this study.(qRT-PCR primers).

Name	Sequences (5'-3')
eEF-1α-F	GCACGCTCTTCTTGCTTTC
eEF-1α-R	AGGGAATCTTGTCAGGGTTG
OsIQM1-F	GCCTGCCTAAACATCAAGTCC
OsIQM1-R	CTCCTACATCCAGCCAGTAAAAG
OsIQM2-F	CTTCTACTGGTTGGATGTGGGT
OsIQM2-R	TGGAGTGCTGGAATGTGCC
OsIQM3-F	GGCACAGAAACTCGCATTACA
OsIQM3-R	CTCGCAGTGGAGCCAGGTA
OsIQM4-F	ATAGTGTCTTCCAGCCCCAAAT
OsIQM4-R	TCAGCGATGCAAAGTCCAAT
OsIQM5-F	CAAGAACACGAGCAGCCAAGGT
OsIQM5-R	GCAGCCAGCAGTCATAGTAGTAGT
OsIQM6-F	CATTGAGAAGCCCGAAACG
OsIQM6-R	TCACCAACATCCAACCAGTAGA
OsIQM7-F	TGTCGTACAGCACCATCTCCTTCC
OsIQM7-R	ACCCTGCCTTGGCGTCTCTTGAT
OsIQM8-F	CCTCCTCCCTCCACCTTAAAC
OsIQM8-R	ATCCAACGCTTGCCACCAG

Vector construction primers

Name	Sequences (5'-3')
Y2H*OsIQM1*-F	CAGTGAATTCCACCCGATGACTCTGCGGCCACTCAAC
Y2H*OsIQM1*-R	TATCGATGCCCACCCTCACTTCCCAAGCTTTGGGAGTGT
Y2H*OsIQM2*-F	CAGTGAATTCCACCCGATGGGCCTGTCAATCTCATAC
Y2H*OsIQM2*-R	TATCGATGCCCACCCTCATTAGGCTGCACCGTGCTGC
Y2H*OsIQM3*-F	CAGTGAATTCCACCCGATGGGCGTCCTCTTCTCGTG
Y2H*OsIQM3*-R	TATCGATGCCCACCCTCAGTCAGCCGCAGCGACGGT
Y2H*OsIQM4*-F	CAGTGAATTCCACCCGATGGGATTGCATCAGCATGC
Y2H*OsIQM4*-R	TATCGATGCCCACCCTCACTGCAATCTGCTCCTCTTG
Y2H*OsIQM5*-F	CAGTGAATTCCACCCGATGGGGGTGCTCTTCTCGTG
Y2H*OsIQM5*-R	TATCGATGCCCACCCTCACCTGATCAGCACTCCCTGCTG

Y2H*OsIQM6*-F	CAGTGAATTCCACCCGATGGGGCTGTCAATCTCAT
Y2H*OsIQM6*-R	TATCGATGCCCACCCTCATTAGAAAGCAGTCCAAGT
Y2H*OsIQM7*-F	CAGTGAATTCCACCCGATGGCGGTGGCGACGGCG
Y2H*OsIQM7*-R	TATCGATGCCCACCCTTAGATACTCGTTGCCGGTTCT
Y2H*OsIQM8*-F	CAGTGAATTCCACCCGATGGGGGTGGACGCTGCT
Y2H*OsIQM8*-R	TATCGATGCCCACCCCTAACTTGGCTGGGGCAGTGAAG
Y2H*OsCaM1*-F	CAGTGAATTCCACCCGATGGCGGACCAGCTCACC
Y2H*OsCaM1*-R	TATCGATGCCCACCCTCACTTGGCCATCATGACC
OsIQM1GFP-F	GAGCTGCAGAAGCTTACTAGTATGACTCTGCGGCCACTCA
OsIQM1GFP-R	TCCACTTCCACCTCCGGTACCCTTCCCAAGCTTTGGGAGTGT
OsIQM2GFP-F	GAGCTGCAGAAGCTTACTAGTATGGGCCTGTCAATCTCATAC
OsIQM2GFP-R	TCCACTTCCACCTCCGGTACCTTAGGCTGCACCGTGCTGC
OsIQM3GFP-F	GAGCTGCAGAAGCTTACTAGTATGGGCGTCCTCTTCTCGTG
OsIQM3GFP-R	TCCACTTCCACCTCCGGTACCGTCAGCCGCAGCGACGGT
OsIQM4GFP-F	GAGCTGCAGAAGCTTACTAGTATGGGATTGCATCAGCATGC
OsIQM4GFP-R	TCCACTTCCACCTCCGGTACCCTGCAATCTGCTCCTCTTG
OsIQM5GFP-F	GAGCTGCAGAAGCTTACTAGTATGGGGGTGCTCTTCTCGTG
OsIQM5GFP-R	TCCACTTCCACCTCCGGTACCCCTGATCAGCACTCCCTGCTG
OsIQM6GFP-F	GAGCTGCAGAAGCTTACTAGTATGGGGCTGTCAATCTCAT
OsIQM6GFP-R	TCCACTTCCACCTCCGGTACCTTAGAAAGCAGTCCAAGT
OsIQM7GFP-F	GAGCTGCAGAAGCTTACTAGTATGGCGGTGGCGACGGCG
OsIQM7GFP-R	TCCACTTCCACCTCCGGTACCGATACTCGTTGCCGGTTCTAACTT
OsIQM8GFP-F	GAGCTGCAGAAGCTTACTAGTATGGGGGTGGACGCTGCT
OsIQM8GFP-R	TCCACTTCCACCTCCGGTACCACTTGGCTGGGGCAGTGAAG
